# Intestinal Immune Cell Populations, Barrier Function, and Microbiomes in Broilers Fed a Diet Supplemented with *Chlorella vulgaris*

**DOI:** 10.3390/ani13142380

**Published:** 2023-07-21

**Authors:** Ji Young Lee, June Hyeok Yoon, Su Hyun An, In Ho Cho, Chae Won Lee, Yun Ji Jeon, Sang Seok Joo, Byeong Cheol Ban, Jae-Yeong Lee, Hyun Jung Jung, Minji Kim, Z-Hun Kim, Ji Young Jung, Myunghoo Kim, Changsu Kong

**Affiliations:** 1 Department of Animal Science, College of Natural Resources & Life Science, Pusan National University, Miryang 50463, Republic of Korea; wldud15873@naver.com (J.Y.L.); ssjoo7680@gmail.com (S.S.J.); banbyeongcheol@gmail.com (B.C.B.); 2 Department of Animal Science and Biotechnology, Kyungpook National University, Sangju 37224, Republic of Korea; junehyeokyoon@gmail.com (J.H.Y.); woobi89@gmail.com (S.H.A.); inhoblog970630@gmail.com (I.H.C.); chaewon2991@gmail.com (C.W.L.); cecil0706@naver.com (Y.J.J.); 3 Animal Genetic Resources Research Center, National Institute of Animal Science, Rural Development Administration, Wanju 55365, Republic of Korea; jay0i@korea.kr; 4 Animal Nutrition and Physiology Team, National Institute of Animal Science, Rural Development Administration, Wanju 55365, Republic of Korea; hyjjung@korea.kr (H.J.J.); mjkim00@korea.kr (M.K.); 5 Microbial Research Department, Nakdonggang National Institute of Biological Resources (NNIBR), Sangju 37242, Republic of Korea; kimzhun@gmail.com (Z.-H.K.); jyjung@nnibr.re.kr (J.Y.J.); 6 Life and Industry Convergence Research Institute, Pusan National University, Miryang 50463, Republic of Korea; 7 Department of Animal Science, Kyungpook National University, Sangju 37224, Republic of Korea; 8 Research Institute of Horse Industry, Kyungpook National University, Sangju 37224, Republic of Korea

**Keywords:** microalgae, immune cells, gut health, microbiome, broilers

## Abstract

**Simple Summary:**

Microalgae have a broad nutritional composition of proteins, lipids, and bioactive compounds such as polysaccharides, carotenoids, and chlorophyll. Even at low inclusion levels, Chlorella vulgaris (CV) is a promising sustainable feed additive because of its positive effects on the nutritional and functional properties of broiler chickens. These features may play a vital role in the early stage of birds as intestinal and immune maturation can lead to improved gut development and production in later stages. Therefore, in this study, we investigated whether incorporating CV into the diet of 10-day-old broilers would enhance their growth performance, gut functional characteristics, and bacterial communities.

**Abstract:**

This study aimed to evaluate the effects of dietary *Chlorella vulgaris* (CV) on the distribution of immune cells, intestinal morphology, intestinal barrier function, antioxidant markers, and the cecal microbiome in 10-day-old broiler chickens. A total of 120 day-old Ross 308 male broiler chicks were assigned to two dietary treatments using a randomized complete block design, with body weight as the blocking factor. Birds fed a diet containing CV showed an increase in CD4^+^ T cells (*p* < 0.05) compared to those fed the control diet. The relative mRNA expression of intestinal epithelial barrier function-related markers (occludin and avian β-defensin 5) was elevated (*p* < 0.05) in the CV-supplemented group compared to the control group. The alpha diversity indices (Chao1 and observed features) of the cecal microbiome in 10-day-old birds increased (*p* < 0.05), indicating higher richness within the cecal bacterial community. In the microbiome analysis, enriched genera abundance of *Clostridium ASF356* and *Coriobacteriaceae CHKCI002* was observed in birds fed the diet containing CV compared to those fed the control diet. Taken together, dietary CV supplementation might alter intestinal barrier function, immunity, and microbiomes in 10-day-old broiler chickens.

## 1. Introduction

The growing global population and rapidly rising incomes have led to an overall increase in the demand for animal-derived products, which account for 40% of total protein consumption [1]. Humans and livestock compete for resources, as high-quality plant protein sources for animal feed have a balanced nutritional value for direct human consumption [2]. Additionally, a major constraint on economical poultry production is the threat posed by pathogens, which can significantly reduce profitability by reducing growth performance and increasing mortality. With the prohibition of antibiotics in the animal feed industry, several studies have been conducted to identify alternatives with antimicrobial, growth-promoting, and immune-stimulating functions. Most previous studies on functional feeds have been conducted to determine their growth-promoting effects on livestock. However, recent studies have focused on the immune regulatory effects of functional diets owing to the importance of animal health. Feed additives thus developed can play an important role in the sustainability of livestock production systems by improving the nutrient efficiency and gastrointestinal health of animals.

Microalgae are an important aquatic resource. The use of microalgae as animal feed, mainly for poultry, is considered a sustainable and promising alternative for addressing the challenges facing the poultry industry [3]. Microalgal photosynthesis has the potential to produce valuable compounds and energy because it utilizes solar energy 50 times more efficiently than terrestrial plants [4]. Among plants with the ability to fix carbon dioxide, microalgae are the most efficient and can convert it into many useful compounds [5]. Supplementation of animal feed with microalgae can provide proteins, lipids, vitamins, minerals, balanced essential amino acids, polysaccharides, and pigments (i.e., carotenoids and chlorophylls) [6]. Therefore, intensive research is being conducted on microalgae, which are known to have beneficial effects on immune response, gut function, antibacterial activity, and intestinal microflora [7].

Although large variations in chemical composition have been reported among different algal samples, the high protein content of different microalgal species is a compelling factor for their application in feed production [8]. For example, the microalgae *Chlorella* has a protein content of 51–58%, and essential amino acids account for approximately 38% of the total amino acids [9,10]. *Chlorella* extracts have been found to have diverse effects, such as promoting growth performance and immune modulation in livestock. Oh et al. reported that the incorporation of 0–0.2% *Chlorella* species into the diet linearly increased the average daily gain, feed intake, and tibia bone strength of male Pekin ducks [11]. The beneficial effects of *Chlorella vulgaris* (CV) have been documented; it improves meat quality by increasing the yellowness of meat due to the high concentration of carotenoids [12]. The effects of CV supplementation on immunoglobulins and inflammatory cytokines have been studied to identify immune-modulatory properties [13,14]. Supplementation of broilers with CV enhances humoral immune responses, such as increased serum levels of IgG and IgM [15].

Nevertheless, extensive studies have been conducted, though there are limited studies on the distribution of immune cells, intestinal health, and bacterial communities in birds fed diets containing CV [16]. Moreover, as young broilers during the first 1–2 weeks after hatching have less diverse microbial communities and more immature immune systems than older birds [17], the supplemental effects of CV on gut immunity and the microbiome need to be established at an early stage. Therefore, the present study aimed to investigate the immune cell profiles, intestinal morphology, barrier functions, serum antioxidant enzyme activities, and cecal microbiomes of 10-day-old broiler chickens fed a CV-supplemented diet.

## 2. Materials and Methods

The experimental procedures were reviewed and approved by the Kyungpook National University Institute for Animal Care and Use Committee of the Republic of Korea (approval number: KNU 2021-0213).

### 2.1. Microalgae Preparation

*Chlorella vulgaris* (FBCC-A49) was locally isolated from the Sangju Weir in Nakdong River (Sangju, Republic of Korea) and stored in the Freshwater Bioresources Culture Collection of Nakdonggang National Institute of Biological Resources (Sangju, Republic of Korea). To attain a high cell concentration (~5 g/L), CV cells were cultivated in N, P-fortified BG11 medium containing 4.25 g/L of NaNO_3_, 0.59 g/L of K_2_HPO_4_, 0.075 g/L of MgSO_4_∙7H_2_O, 0.036 g/L of CaCl_2_∙2H_2_O, 0.006 g/L of citric acid, 0.006 g/L of ferric ammonium citrate, 0.001 g/L of ethylenediaminetetraacetic acid, 0.02 g/L of Na_2_CO_3_, and 1 mL of trace metal mix A5 solution consisting of 2.86 g/L of H_3_BO_3_, 1.81 g/L of MnCl_2_∙4H_2_O, 0.222 g/L of NaMoO_4_∙2H_2_O, 0.079 g/L of CuSO_4_∙5H_2_O, and 0.05 g/L of CoCl_2_∙6H_2_O [18]. Seed cultures were maintained in 2.5 L bubble column photobioreactors (BC-PBRs) containing 2 L of fresh medium, incubated at 22 ± 1 °C, and irradiated at 300 µE/m^2^/s using 55 W fluorescent lamps with 2% (*v*/*v*) continuous CO_2_ bubbling at 0.1 vvm. Cultures were then scaled up to 100 L flat-plate PBRs under the same conditions as those used for the BC-PBRs. CV cells were harvested using a continuous centrifuge (J1250, Hanil Science Co., Ltd., Daejeon, Republic of Korea) at 13,700× *g*. For freeze drying, collected wet biomass was frozen overnight at −80 °C and lyophilized using a freeze dryer (FD8512, IlShinBioBase Co., Ltd., Dongducheon, Republic of Korea). Dried cells were stored in a desiccator until used in experiments.

### 2.2. Animals, Diets, and Experimental Design

A total of 120 day-old male Ross 308 broilers with an initial body weight (BW) of 39.43 ± 2.7 g were used for this experiment. All birds were individually weighed and tagged with identification numbers. The birds were allocated to two dietary treatments in a randomized complete block design with initial BW as the blocking factor. Each dietary treatment consisted of six replicate cages with 10 birds per cage. The two experimental diets were corn–soybean meal-based diets supplemented with microalgae (CV) at 0% or 0.5%, respectively (Table 1). The experimental diets were formulated to meet or exceed the nutrient recommendations described in the Nutritional Specifications for Ross 308 Male Broilers. The diets were provided in the mashed form, and each cage had a drinker connected to a water source via two nipples. The birds had ad libitum access to feed and water throughout the experimental period. The feeding period of the present study was 10 days to investigate the effects of CV supplementation on the early stage of broilers. Birds were housed in battery cages (60 × 50 × 60 cm^3^) in a controlled room under continuous lighting. The room’s temperature was 34 °C on the first day and gradually decreased by 2 °C per week until day 10. On day 9, the individual BW of the birds and feed leftovers were weighed to measure BW gain, feed intake (FI), and gain-to-feed ratio (G:F).

### 2.3. Sample Collection

On day 10, the birds were euthanized via CO_2_ asphyxiation. Blood was collected from the jugular vein along with intact jejunal tissue (10 cm of the central part of the jejunum) and cecal digesta from the median BW birds in each cage. The jejunal tissue was cut into 4 cm pieces for jejunal immune cell isolation, a 1 cm piece for jejunal histology, and a 0.5 cm piece for RNA extraction. For jejunal histology, a 1 cm section of jejunal tissue was fixed in 10% neutral buffered formalin for 48 h. The remaining 1 out of 9 birds per cage were dissected to collect cecal digesta, which was stored at −80 °C.

### 2.4. Jejunal Lamina Propria Isolation

Lamina propria immune cells were isolated from jejunal tissue and cut into 4 cm sections. The tissues were transferred to a petri dish with cold PBS to remove all mesenteric material and fat. The intestinal tissue was opened transversely and flushed with PBS. The mucus layer was scraped off using a coverslip. The tissues were then treated with 1 mM DL-dithiothereitol (DTT; Sigma-Aldrich, Irvine, UK), 30 mM ethylenedia-mine-tetraacetic acid (EDTA; Thermo Fisher Scientific/Ambion, Waltham, MA, USA), and 10 mM 4-[2-hydroxyethyl]-1-piperazineethanesulfonic acid (HEPES; Thermo Fisher Scientific, Waltham, MA, USA) in PBS for 10 min at 37 °C in a shaking incubator (200 rpm) to remove the mucus and epithelium. After incubation, tissues were washed with PBS for 1 min, treated with 30 mM EDTA and 10 mM HEPES in PBS for 10 min at 37 °C in a shaking incubator (200 rpm), and washed with PBS for 1 min. The tissues were transferred to complete RPMI 1640 medium (cRPMI; Gendepot, Barker, TX, USA) with 10% fetal bovine serum (FBS; Gibco BRL, Burlington, ON, Canada) and 1% penicillin–streptomycin (P/S; Hyclone Laboratories, Logan, UT, USA) and washed gently for 2 min. They were digested with collagenase type VIII (Sigma-Aldrich, St. Louis, MO, USA) in cRPMI for 1 h at 37 °C in a shaking incubator (200 rpm). They were then centrifuged (800× *g*, 5 min, RT), the supernatant was removed, and the lamina propria cells were separated using the concentration difference of Percoll (Cytiva, Marlborough, MA, USA). Cells collected from the Percoll layer were used for flow cytometry.

### 2.5. Flow Cytometry Analysis

The cells isolated from the jejunum were stained for flow cytometry. The LIVE/DEAD™ Fixable Aqua Dead Cell Stain Kit (Invitrogen, Thermo Fisher Scientific, Seoul, Republic of Korea) was used to analyze the live cells. The stain was diluted 1:1000 in PBS and stained with other antibodies. Macrophages and B cells were stained with PE-conjugated mouse anti-chicken Mono/Macro (8420-09, Southern Biotech, Birmingham, AL, USA), FITC-conjugated mouse anti-chicken MHCII (8350-02, Southern Biotech, Birmingham, AL, USA), and Alexa Fluor 647-conjugated mouse anti-chicken Bu-1 antibodies (8395-31, Southern Biotech). T cell subsets were stained with Pacific Blue-conjugated mouse anti-chicken CD3 (8200-26, Southern Biotech, Birmingham, AL, USA), FITC-conjugated mouse anti-chicken CD4 (8210-02, Southern Biotech, Birmingham, AL, USA), SPRD-conjugated mouse anti-chicken CD8α (8220-13, Southern Biotech, Birmingham, AL, USA), and PE-conjugated mouse anti-chicken TCRγδ antibodies (8230-09, Southern Biotech, Birmingham, AL, USA). Each antibody was diluted 1:200 in PBS and used for staining. The cells were measured using an FACS Canto II (Becton Dickinson, Heidelberg, Germany) and analyzed using FlowJo software v10.7.1 (Tree Star Inc., Ashland, OR, USA).

### 2.6. RNA Extraction and Reverse Transcription

The jejunum tissue (0.5 cm) was processed using a Bioprep-6 homogenizer (Bioand, Namyangju, Republic of Korea). It homogenized at 6 m/s for 30 s and rested on ice for 1 minute, repeated two times. The homogenate was centrifuged for 10 min at 10,000× *g* at 4 °C and the supernatant was mixed with chloroform (chloroform:isoamylalcohol (24:1), Biosesang, Seongnam, Republic of Korea). The mixture was incubated for 3 min at room temperature and centrifuged for 20 min at 10,000× *g* at 4 °C. The supernatant was transferred to a tube containing isopropanol (Biosesang, Seongnam, Republic of Korea), gently mixed, and incubated for 10 min at room temperature. The sample was centrifuged for 10 min at 10,000× *g* at 4 °C, the supernatant was removed, and 75% DEPC-EtOH made from DEPC-treated water (Invitrogen, Carlsbad, CA, USA) and EtOH was added to it (Biosesang, Seongnam, Republic of Korea). This mixture was centrifuged for 5 min at 7500× *g* at 4 °C and the supernatant was removed. The pellet was dried and dissolved in DEPC-treated water. RNA quality and concentration were checked using a microspectrophotometer (AllSheng Instrument Inc., Hangzhou, China). cDNA was prepared using AccuPower RT PreMix (Bioneer, Daejeon, Republic of Korea).

### 2.7. Quantitative Real-Time PCR Analysis

cDNA was prepared using SologTM h-Taq DNA polymerase (SolGent, Daejeon, Korea) for amplification. Gene expression was measured using QuantStudio1 (Applied Biosystems, Waltham, CA, USA). The program proceeded with a hold stage (50 °C for 2 min, 95 °C for 10 min), PCR stage (95 °C for 30 s and 60 °C for 30 s; 40 cycles) followed by a melt curve stage (60 °C for 1 min, 95 °C for 1 s). The results were analyzed using the ΔΔCt method. The primers used for gene amplification were as follows: glyceraldehyde 3-phosphate dehydrogenase (GAPDH) (F: GTCCTCTCTGGCAAAGTCCAAG, R: TCACAAGTTTCCCGTTCTCAGC), occludin (OCLN) (F: GATGGACAG-CATCAACGACC, R: CTTGCTTTGGTAGTCTGGGC), mucin 2 (MUC2) (F: GGGA-TATTCACCTGGAGAAACCATT, R: TTGCATGTCCATCTGCCTGAA), avian β-defensin (AvBD5) (F: GAGCCGATGGTATTCCTGATGG, R: GTGGTGATTGTT-GCCTCTGGTG), superoxide dismutase (SOD1) (F: AGGGGGTCATCCACTTCC, R: CCCATTTGTGTTGTCTCCAA), and catalase (CAT) (F: TTACGGAGGTAGAACAGATGG, R: TGTCAGGATACGCAAAGAGA).

### 2.8. Serum Antioxidant Enzyme Analysis

The serum samples were collected via centrifugation at 2000× *g* for 15 min at RT and were then stored at −20 °C before the analysis of antioxidant enzymes. The serum samples were used to determine the concentrations of malondialdehyde (MDA; Cell Biolabs, Inc., San Diego, CA, USA), nitric oxide (NO; BioAssay Systems, Hayward, CA, USA), catalase (CAT; Cell Biolabs, Inc., San Diego, CA, USA), glutathione peroxidase (GPx; BioAssay Systems, Hayward, CA, USA), and superoxide dismutase (SOD; BioAssay Systems, Hayward, CA, USA) according to the manufacturer’s instructions.

### 2.9. Jejunal Histology

Tissues separated from the small intestine were cut into 1 cm pieces and fixed with formalin solution (Sigma-Aldrich) before hematoxylin and eosin (H&E) staining. Paraffin blocks were then prepared for H&E staining at Pusan National University’s Image Core Lab (Yangsan, Republic of Korea). The fixed tissue was embedded in a paraffin block, cut into cross sections, and stained with H&E. Villus height and crypt length were measured under a microscope.

### 2.10. Cecal Short-Chain Fatty Acids Analysis

Approximately 0.5 g of cecal digesta was suspended in 4.5 mL of cold distilled water and supplemented with 0.025 mL of saturated mercury (II) chloride and 0.5 mL of 25% metaphosphoric acid to stop microbial metabolism, with 0.1 mL of 2% pivalic acid then added as a reference substance for the measurement of short-chain fatty acids [19]. The cecal digesta samples were then centrifuged at 1000× *g* at 4 °C for 20 min, and 1 mL of the supernatant was collected to measure the concentration of short-chain fatty acids in the digesta. The samples were injected into a gas chromatography system (6890 Series GC System, HP, Palo Alto, CA, USA) equipped with a flame ionization detector and a capillary column (30 m × 0.25 mm × 0.25 μm; Agilent, Santa Clara, CA, USA) operated at 50 °C in the oven. The inlet and detector temperatures were set at 180 °C and 250 °C, respectively. Helium was used as the carrier gas.

### 2.11. Cecal Microbiome Analysis

DNA was quantified using the DNeasy PowerSoil Kit (Qiagen, Hilden, Germany) according to the protocol in the DNeasy PowerSoil Kit Handbook, and the extracted DNA was quantified using Quant-IT PicoGreen (P7589, Invitrogen). Subsequent intestinal microbial analysis was conducted following the 16S Metagenomic Sequencing Library protocol provided by Illumina Inc. For amplicon PCR, the V3-V4 region of 16S rRNA genes was targeted, and the primers used were as follows: V3: 5′-TCG TCG GCA GAT GTG TAT AGA CAG CCTG CGN GGC WGC AG-3′ (forward primer) and V4: 5′-GTCTCGGGGGGGGGTAGAGAGAGAGAGAGAGAGACHVGTATATCC-3′ (reverse primer). The manufactured library was entrusted to Macrogen for Miseq (Illumina, San Diego, CA, USA). The sequence reads were quality-filtered and denoised, and the paired-end sequence data were merged using the DADA2 (ver. 1.18) plugin. Additionally, the chimeric sequences were then removed. For the paired-end reads, the forward and reverse sequences were cut into 251 bp and 227 bp, respectively. The sequences were assembled using QIIME2 (ver. 2022.2) and the intestinal microbial clusters were compared. Based on the amplicon sequence variant level, alpha diversity was analyzed using the Shannon index, reverse Simpson index, and Chao1, and these were calculated from rarefied abundance tables using 54,427 sequences per sample. A linear discriminant analysis (LDA) score of 2.0 or higher was used as an effect size index using linear discriminant analysis (LEfSe).

### 2.12. Statistical Analysis

Data were processed using the TTEST procedure in Prism (GraphPad, La Jolla, CA, USA). Significance was declared at less than 0.05.

## 3. Results

### 3.1. Growth Performance

The effects of CV supplementation on the growth performance of 10-day-old broilers fed either a control diet or a diet containing CV are shown in Table 2. The BW gain, FI, and G:F of birds fed the diet containing CV did not differ from those of birds fed the control diet. Outliers, defined as values outside of ±2.0 × interquartile range, were removed from the growth performance data.

### 3.2. Jejunal Morphology

**Figure 1:**
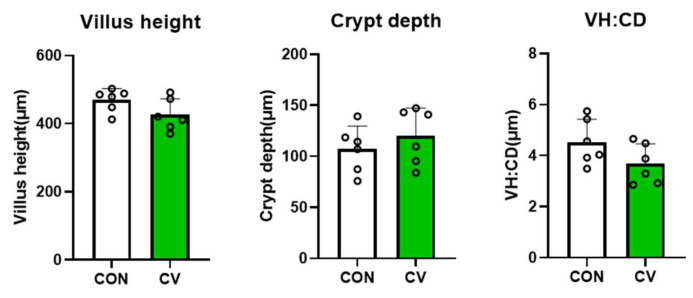
Villus height (VH), crypt depth (CD), and VH:CD of 10-day-old broilers fed a control diet or a *Chlorella vulgaris* (CV)-containing diet. Each data point represents six replicates and is presented as mean ± standard deviation. Significant differences between the treatments were analyzed using the 1-sample 2-tailed *t*-test.

The effects of CV on the jejunal morphology of broiler chickens fed a CV-supplemented diet on day 10 are summarized in Figure 1. Villus height, crypt depth, and the villus height to crypt depth ratio (VH:CD) were not affected by CV supplementation in the broiler diet.

### 3.3. Relative mRNA Expression of Antioxdant Enzymes in Jejunal Tissues and Antioxidant Enzyme Concentrations in Serum

**Figure 2:**
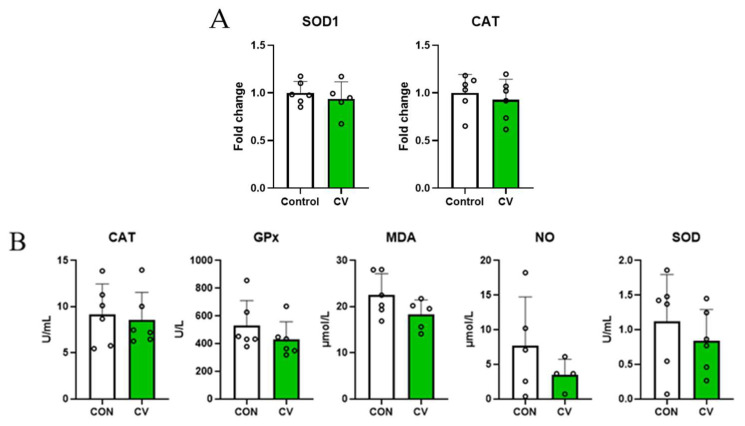
(**A**) Relative mRNA expression of antioxidant markers in jejunal tissues and (**B**) concentrations of antioxidant enzymes in the serum of 10-day-old broilers fed a control diet or a *Chlorella vulgaris* (CV)-containing diet. Each data point represents six replicates and is presented as mean ± standard deviation. Significant differences between the treatments were analyzed using the 1-sample 2-tailed *t*-test.

The mRNA expressions of antioxidant markers in jejunal tissues and antioxidant enzyme concentrations in the serum of 10-day-old broilers fed a diet containing CV are shown in Figure 2. The relative mRNA expression levels of antioxidant enzymes and antioxidant enzyme activities in birds fed the diet containing CV did not differ from those in birds fed the control diet.

### 3.4. Relative mRNA Expression of Intestinal Epithelial Barrier Functions in Jejunal Tissues

**Figure 3:**
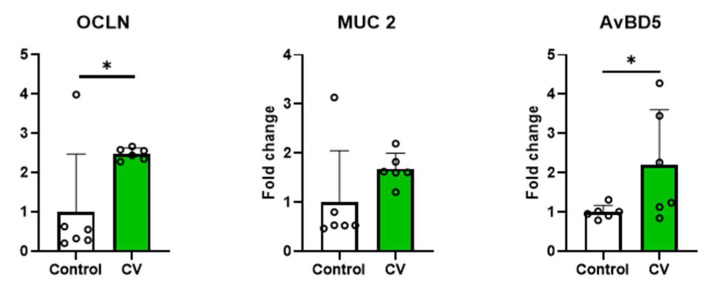
Effects of dietary *Chlorella vulgaris* (CV) supplementation on the relative mRNA expression of occludin (OCLN), mucin 2 (MUC2), and avian β-defensin 5 (AvBD5) in the jejunal tissue of 10-day-old broiler chickens. Each data point represents six replicates and is presented as mean ± standard deviation. Significant differences between the treatments were analyzed using the 1-sample 2-tailed *t*-test. * *p* < 0.05.

The effects of dietary CV supplementation on the relative jejunal mRNA expression of tight junction protein (OCLN), mucus (MUC2), and antimicrobial peptides (AMP; AvBD5) in 10-day-old broiler chickens are summarized in Figure 3. The mRNA expression of OCLN and AvBD5 genes in jejunal tissues of birds fed the CV-supplemented diet was higher (*p* < 0.05) than it was in birds fed the control diet.

### 3.5. Immune Cell Populations in Jejunal Lamina Propria

**Figure 4:**
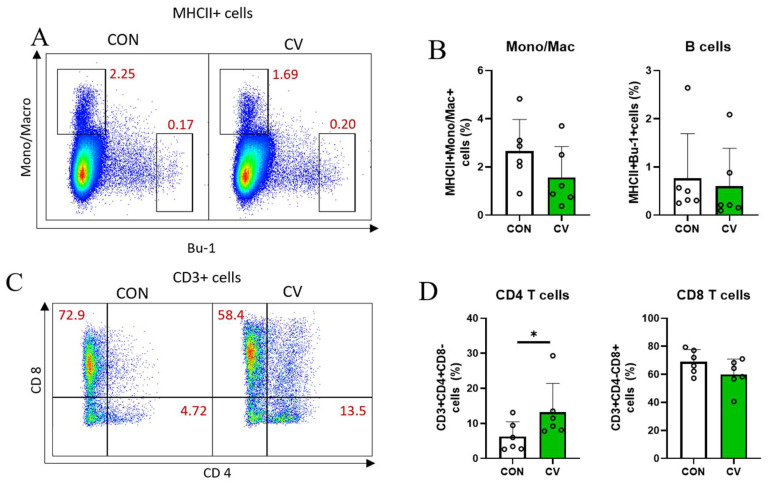

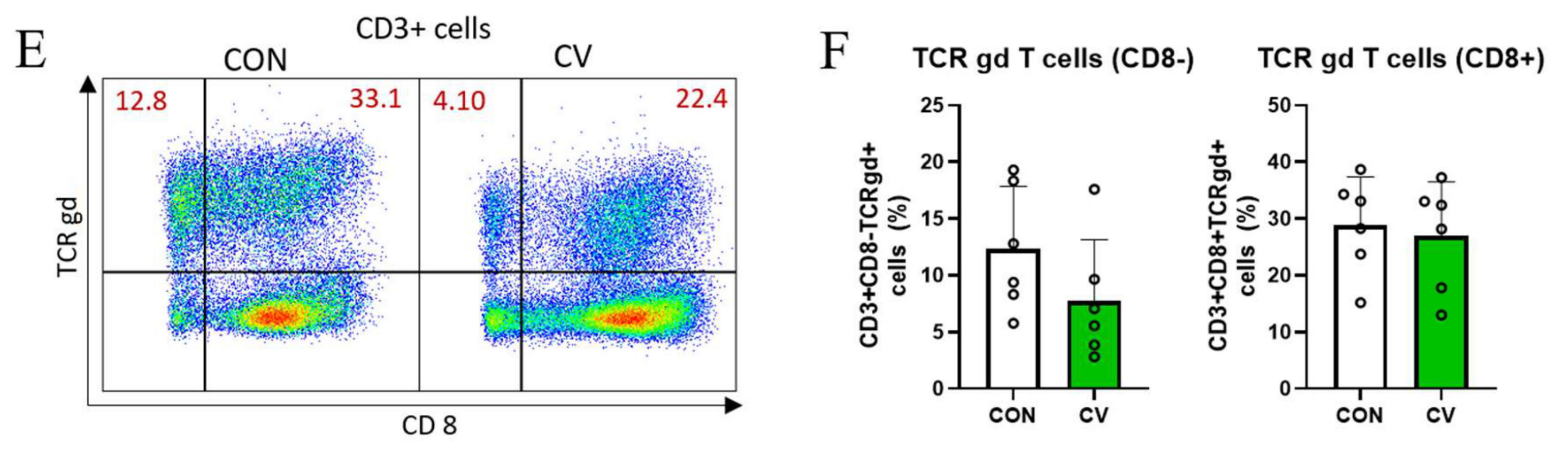
Changes in the populations of immune cells in the jejunal lamina propria of 10-day-old broilers fed a control diet or a *Chlorella vulgaris* (CV)-containing diet. (**A**) The distribution of monocyte/macrophages (Mono/Macro) and B cells in MHCII cells; (**B**) populations of Mono/Macro and B cells in MHCII cells; (**C**) the distribution of CD8 and CD4 cells in CD3 cells; (**D**) populations of CD4 and CD8 T cells in CD3 cells; (**E**) the distribution of TCRγδ and CD8 cells in CD3 cells; (**F**) populations of TCRγδ T cells in CD3 cells. In figure (**A**,**C**,**E**), colors represent the density of cells in that location and indicate the density of cells in the order of red, yellow, green, and blue. Each data point represents six replicates and is presented as mean ± standard deviation. Significant differences between the treatments were analyzed using the 1-sample 2-tailed *t*-test. * *p* < 0.05.

Flow cytometry analysis was conducted to determine the proportion of immune cells in the jejunal lamina propria of 10-day-old broiler chickens fed the experimental diets. For innate immune cells, antigen-presenting cells (APCs) were identified using an antigen for MHCII. Among the APCs (MHCII^+^ cells), macrophages and B cells were identified based on Mono/Mac and Bu-1 expression, respectively. The total APC population was not affected by CV supplementation. In addition, there were no differences between macrophages (MHCII^+^ Mono/Mac^+^ cells) and B cells (MHCII^+^Bu-1^+^ cells; Figure 4A,B). For adaptive immune cells, T cells were identified using an anti-CD3 antibody. Most of the T cells in the small intestine were CD8^+^ T cells (~80%). CV supplementation increased (*p* < 0.05) in CD4^+^ T cell populations but did not affect CD8^+^ T cell populations in the small intestine of broiler chickens (Figure 4C,D). The subsets of TCRγδ T cells, such as CD3^+^CD8^+^TCRγδ T cells and CD3^+^CD8^−^TCRγδ T cells, were not affected by CV supplementation (Figure 4E,F).

### 3.6. Cecal Microbiome

**Figure 5:**
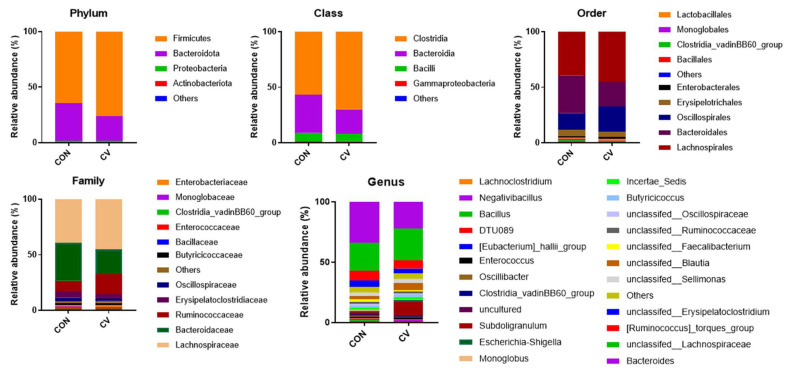
Effects of *Chlorella vulgaris* (CV) supplementation on the phylum, class, order, family, and genus-level compositions of cecal microbial communities in 10-day-old broilers. All levels that accounted for less than 1% of the bacterial taxonomic abundance were classified into the “Others” category. Each color represents a different taxonomic unit (*n* = 6).

The V3–V4 regions of the 16S rRNA genes were sequenced to investigate the composition and diversity of cecal bacterial communities in birds fed the control and CV-supplemented diets. The average relative abundances of cecal microbial communities in birds fed the CV-supplemented diet are shown in Figure 5. At the phylum level, the control group was predominantly composed of *Firmicutes* (64.66%), *Bacteroidota* (34.00%), and *Proteobacteria* (1.19%). The CV-supplemented group consisted primarily of *Firmicutes* (76.33%), *Bacteroidetes* (34.00%), and *Proteobacteria* (1.19%). CV supplementation increased the abundance of *Firmicutes* in the CV group and decreased the abundance of *Bacteroidetes* compared to the control group. However, the ratio of *Firmicutes* to *Bacteroidetes* was consistent (98.65% and 98.42%) across the dietary treatments. At the genus level, *Bacteroides* was the most enriched genus in the cecal bacterial communities of 10-day-old birds. The relative abundance of *Bacteroides* in birds fed the CV-supplemented diet increased by 11.91% compared to birds fed the control diet. However, the relative abundance of the *Lachnospiraceae* family decreased by 3.40% in the CV-supplemented group compared to the control group.

**Figure 6:**
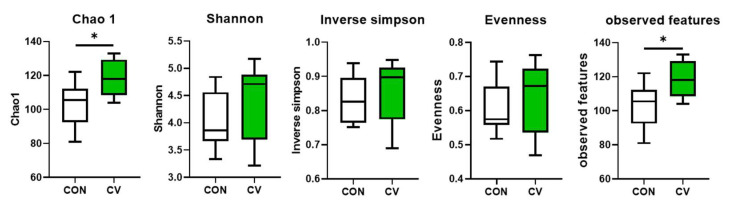
Effects of dietary *Chlorella vulgaris* (CV) supplementation on the alpha diversity indices of the cecal microbial communities in 10-day-old broilers. Each data point represents six replicates. Significant differences between the treatments were analyzed using the 1-sample 2-tailed *t*-test. * *p* < 0.05.

The alpha diversity indices of the cecal microbiome in the control and CV-supplemented groups are shown in Figure 6. No differences in alpha diversity metrics (Shannon, Inverse Simpson, and evenness) were observed among the dietary treatments on day 10. However, the Chao1 index and the observed features in birds fed the CV-supplemented diet were greater (*p* < 0.05) than those in birds fed the control diet.

**Figure 7:**
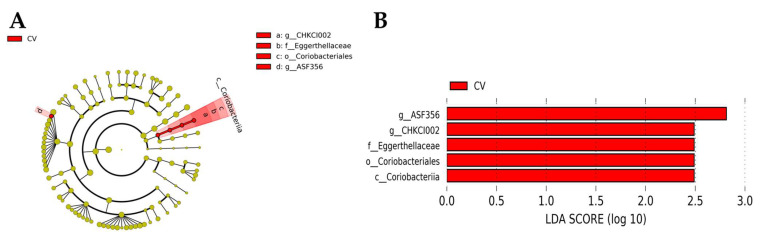
Linear discriminant analysis effect size (LEfSe) and linear discriminant analysis (LDA) based on operational taxonomic units were used to differentiate the cecal microbial communities of birds fed a control diet or a *Chlorella vulgaris* (CV)-containing diet. (**A**) Cladogram generated using LEfSe indicating the phylogenetic distribution of the cecal microbiome in 10-day-old broilers fed a diet containing CV. (**B**) Histogram of LDA scores indicating differences in the cecal microbiome of 10-day-old broilers fed a control diet or a CV-containing diet.

LEfSe analysis was conducted to investigate differentially abundant ASVs between the control and CV-supplemented groups. In the CV-supplemented birds, LEfSe indicated a greater differential abundance for the class *Coriobacteriia*, order *Coriobacteriales*, family *Eggerthellaceae*, and genera *ASF356* and *CHKCI002* (Figure 7A); their corresponding phylogenetic relationships are shown in Figure 7B.

### 3.7. Concentrations of Short-Chain Fatty Acids in Cecal Digesta

**Figure 8:**
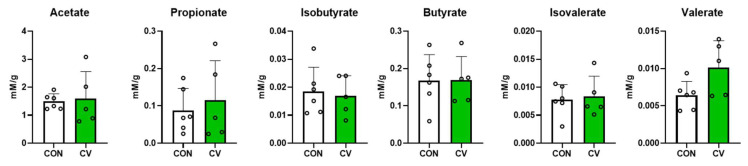
Concentrations of short-chain fatty acids in the cecal digesta of 10-day-old broilers fed a control or a diet containing *Chlorella vulgaris* (CV). Each data point represents six replicates and is presented as mean ± standard deviation. Significant differences between the treatments were analyzed using the 1-sample 2-tailed *t*-test.

The concentrations of short-chain fatty acids in the cecal digesta of broilers fed the control diet or CV-supplemented diet are shown in Figure 8. CV supplementation in the diet of 10-day-old broilers did not affect the concentration of short-chain fatty acids in the cecal digesta.

## 4. Discussion

In the present study, dietary supplementation with 5% CV in 10-day-old broilers did not affect the growth performance of the birds. The beneficial effects of *Chlorella* byproducts on growth performance have been reported in broiler chickens [20,21]. Dietary supplementation with 1% microalgae (CV and *Amphora coffeaformis*) increased the final BW and BW gain of broiler chickens raised from 0 to 36 days [13]. Even at lower inclusion levels (less than 0.5%), the dietary incorporation of CV had a positive effect on the growth performance of birds. An et al. reported that the final BW and daily weight gains of birds fed 0.15% or 0.5% dried *Chlorella* powder were higher than those in the control group [15]. Supplementation of fermented CV in the diet showed linear increases in BW gain and FI in Pekin ducks when CV supplementation was increased from 0% to 0.2% [11]. The discrepancies in the effects of microalgae on growth performance may be attributed to species, habitats, growing conditions (i.e., temperature and light exposure), and collection methods, which result in variable compositions of microalgae [6]. Cabrol et al. demonstrated that birds fed diets containing higher amounts (10–20%) of CV showed reduced BW gain and FI [22]. This may be due to the poor palatability of feed derived from the high inclusion of microalgae. In addition, the high non-starch polysaccharide content of microalgae can increase intestinal viscosity, which reduces feed passage rate and nutrient digestibility, thereby reducing growth performance [23]. Therefore, in the present study, the inclusion rate of CV in the diet was limited to 0.5% to avoid negative impacts on growth performance.

The jejunum is the site where nutrients are decomposed and absorbed, and feed efficiency depends on the morphological characteristics of the intestine [24]. Longer villi increase surface area to support greater nutrient absorption. Newly generated epithelial cells are present in intestinal mucosal crypts [25]. Therefore, deeper crypts indicate greater cell turnover in the villi in response to normal cellular sloughing and the inflammatory response [26]. The VH:CD ratio is used as a criterion to determine the digestive capacity and protective function of animals [27]. Previous studies have shown that VH and CD increase with the supplementation of *Chlorella* byproducts in broiler diets [21,28]. Nevertheless, the results of the present study indicate that dietary supplementation with CV may not improve intestinal morphology in the early stages of broiler chickens. However, Roques et al. noted that CV supplementation may improve broiler performance by alleviating the immune system rather than improving nutrient absorption [29], which is consistent with the findings of the current study.

Oxidative stress occurs because of an imbalance between reactive oxygen species and antioxidant enzyme activity [30]. Antioxidant enzymes (SOD, CAT, and GPx) are the main antioxidant systems that respond to oxidative stress [31]. MDA is one of the end products of lipid oxidation, and excessive lipid peroxidation induces oxidative stress, resulting in MDA accumulation [32]. El-Bahr et al. [13] reported that birds fed a diet containing 1% CV showed increased SOD and decreased MDA concentrations in breast muscle at 32 days, which is supported by the results of [28]. However, in the present study, relative mRNA expression of antioxidant markers and serum antioxidant enzyme activity in birds fed a diet containing CV did not differ from that in birds fed a control diet. Carotenoid pigments, which are present at 8–80 mg/kg in *Chlorella* spp., act as antioxidants [33]. Carotenoids reduce oxidative stress in animals by up-regulating the production of antioxidant enzymes [34]. However, owing to the low inclusion rate (0.5%) of CV in the current study, low concentrations of hepatic carotenoids may not have been sufficient to promote antioxidant enzyme activity [35].

As a part of the mucosal tissue, the intestinal environment is susceptible to the influence of various external antigens, including toxins and pollutants. The intestine serves as a main pathway for microbial infection by pathogens. Barrier function is important to prevent the invasion of intestinal antigens, and inflammation can easily occur when mucosal barrier function deteriorates [36]. In the present study, the expression of OCLN increased in birds fed CV-containing diets. Tight junction proteins are connected to epithelial cells, forming paracellular barriers that regulate the selective permeability of ions, solutes, and nutrients [37]. OCLN, a major component of the tight junction protein complex, plays a crucial role in regulating the diffusion of small molecules across intermembrane and paracellular spaces [38]. An increase in OCLN was not observed in broilers supplemented with another species of microalgae (*Kappaphycus alvarezii*) [39]. However, the aqueous extract of *Chlorella pyrenoidosa* showed an increase in tight junction proteins such as OCLN and Claudin 4 in mice [40]. In the present study, CV supplementation increased the mRNA expression of AvBD5 in broilers. The primary role of AMPs is to kill pathogens by disrupting their cell membranes or inhibiting protein and DNA synthesis. The AMPs also play an important role in maintaining the function of the intestinal barrier, as they stimulate the growth of intestinal epithelial cells and enhance the barrier function of epithelial tissues. Intestinal inflammation often occurs when gut barrier function deteriorates due to weakened tight junctions and the decreased secretion of mucus or AMPs. Therefore, the increased expression of tight junction proteins and AMPs may help fortify gut barrier function. Collectively, the results of the present study suggest that CV supplementation improves the gut barrier function of 10-day-old broilers by up-regulating the gene expression of tight junction proteins, mucus, and AMPs.

Beneath the mucosal barrier is a special layer called the lamina propria, which together with the epithelial cells forms the mucosa. The lamina propria contains several types of immune cells, including lymphocytes, fibroblasts, plasma cells, macrophages, eosinophilic leukocytes, and mast cells. During infection, monocytes are recruited to tissues and differentiate into macrophages [41]. Macrophages, such as APCs, can distinguish between self and non-self, tissue damage, and invading pathogens in the innate immune system [42]. In the present study, no changes were observed in the monocyte/macrophage population following CV supplementation. Adaptive immune cells, especially T cells, play an important role in chronic intestinal inflammation in broiler chickens, as reported by the authors of [43]. CD4^+^ (helper) T cells can differentiate into two types of effector T cells that activate the microbial properties of macrophages and initiate humoral immune responses by activating naïve antigen-specific B cells [44]. Fries-Craft et al. showed that there were augmented CD3^+^CD4^+^ T cell and CD3^+^TCRγδ^+^ T cell populations in the spleen of 14-day-old broilers fed a corn–soybean meal diet containing an algae-based feed ingredient [45]. It has been reported that the development of immune cells in broilers begins to increase on the 7th day of CD4^+^ T cells being present in the peripheral blood and reaches a peak around the 30th day [46]. An increase in CD4^+^ T cells in the ileum has been reported after day 14 [46]. Although the tissue was different from the ileum, our results showed an increase in CD4^+^ T cells in the jejunum even after 10 days. This suggests that the development of CD4^+^ T cells may be promoted by CV supplementation. CD8^+^ (cytotoxic) T cells can kill the infected target cells and pathogens. In the present study, CD4^+^ T cells increased when the diet was supplemented with CV in 10-day-old broilers, suggesting an expansion of helper T cells in the jejunal lamina propria. These results may be partially explained by the microalgal components, including β-carotene, cobalamin, β-glucan, and immunomodulatory polysaccharides, which play vital roles in the immune system and inflammatory processes. The immunomodulatory properties of *Chlorella* species in the postnatal period of broiler chicks are important for driving carry-over effects on intestinal development and immune capability in later stages [47].

The gastrointestinal microbiome plays an important role in gut-specific host immunity and homeostasis. The gut microbiome, which degrades algal polysaccharides or other polymers in animal feed, can increase the digestive efficiency of the host animal [21]. The majority of intestinal microbial communities develop after hatching, with subsequent microbiota composition and activity influenced by numerous host and environmental factors such as host genetics, feed additives, and dietary composition [48]. An important feature of bacterial communities is their diversity, which is characterized by the number and composition of species [49]. The Chao1 index evaluates the total richness of a community in a sample, whereas the Shannon and Simpson indices estimate species richness and evenness [49]. In the present study, an increased Chao1 index and the observed features of birds fed a diet supplemented with CV suggest that the microbial community was enriched compared to that of birds fed the control diet. The LEfSe analysis performed in the present study revealed that *Clostridium ASF356* and *Coriobacteriaceae CHKCI002* were enriched in the CV-supplemented group compared to the control group. *Clostridium ASF356* can produce amino acids (aspartate, lysine, methionine, and phenylalanine) and short-chain fatty acids (propionate and butyrate) [50]. *Coriobacteriaceae CHKCI002*, affiliated with the family *Eggerthellaceae*, has been documented to show positive correlations with butyrate, isobutyrate, valerate, and BW [51]. Short-chain fatty acids are metabolites generated by resident bacterial species for immune responses and anti-inflammatory factors in the gut [52]. Although these microbes are considered as short-chain fatty acid-producing bacteria, they can only produce metabolites in specific growth environments [50]. Therefore, a greater abundance of bacterial species does not necessarily lead to an increase in short-chain fatty acids in the cecal digesta of birds. Thus, further studies are warranted to investigate inconsistencies between the gut microbiome and metabolites in relation to different feed compositions, housing conditions, and production systems.

## 5. Conclusions

In conclusion, dietary CV supplementation increased the gene expression of epithelial barrier functions, augmented CD4^+^ T cell proportions, and diversified the cecal microbiome in 10-day-old broiler chickens. The results of the present study suggest that CV supplementation may have the potential to be a sustainable feed in addition to improving gut barrier functions, immunity, and bacterial communities.

## Figures and Tables

**Table 1:** Ingredient and chemical compositions of experimental diets (as-fed basis).

	Experimental Diets ^1^	
Item	Control	*Chlorella vulgaris*
Ingredient compositions, %		
Corn	53.54	
Soybean meal	38.80	
Cornstarch	0.50	–
Microalgae (*Chlorella vulgaris*)	–	0.50
Soybean oil	2.00	
_L_-arginine	0.09	
_L_-histidine	0.03	
_L_-isoleucine	0.10	
_L_-lysine-HCl	0.35	
_L_-methionine	0.24	
_L_-cysteine	0.14	
_L_-threonine	0.14	
_L_-valine	0.2	
Limestone	1.06	
Dicalcium phosphate	1.91	
Sodium chloride	0.40	
Vitamin premix ^2^	0.20	
Mineral premix ^3^	0.20	
Choline chloride	0.10	
Calculated chemical compositions, %		
MEn, kcal/kg	2976	2972
Crude protein	23.0	23.0
Total calcium	0.96	0.96
Non-phytate phosphorus	0.48	0.48
Calculated amino acids compositions, %		
SID arginine	1.31	
SID histidine	0.47	
SID isoleucine	0.87	
SID leucine	1.53	
SID lysine	1.28	
SID methionine	0.48	
SID cysteine	0.40	
SID phenylalanine	0.92	
SID threonine	0.81	
SID tryptophan	0.23	
SID valine	1.01	

MEn, nitrogen-corrected metabolizable energy; SID, standardized ileal digestible. ^1^ Experimental diets consisted of corn–soybean meal-based diets supplemented with microalgae (*Chlorella vulgaris*) at 0 and 0.5%, respectively. ^2^ The following quantities per kilogram of diet were provided: retinyl acetate, 24,000 IU; cholecalciferol, 8000 IU; _DL_-α-tocopherol acetate, 160 mg; menadione nicotinamide bisulfite, 8 mg; thiamine mononitrate, 8 mg; riboflavin, 20 mg; pyridoxine hydrochloride, 12 mg; _D_-calcium pantothenate, 40 mg; folic acid, 4 mg; nicotinamide, 12 mg. ^3^ The following quantities per kilogram of diet were provided: iron, 120 mg; copper, 320 mg; zinc, 200 mg; manganese, 240 mg; cobalt, 2 mg; selenium, 0.6 mg; iodine, 2.5 mg.

**Table 2:** Growth performance of 10-day-old broilers fed a control diet or a diet containing *Chlorella vulgaris* ^1,2^.

Item	Experimental Diets		SEM	*p*-Value
	Control	*Chlorella vulgaris*		
Body weight gain, g/bird	156.2	150.9	5.38	0.520
Feed intake, g/bird	175.0	169.1	3.27	0.258
Gain to feed ratio, g/g	0.89	0.89	0.016	0.929

^1^ Experimental diets consisted of corn–soybean meal-based diets supplemented with microalgae (*Chlorella vulgaris*) at 0 and 5 g/kg, respectively. ^2^ Data represent six replicate cages, with 10 birds used from day 0 to 10 post hatch. Significant differences between the treatments were analyzed using the 1-sample 2-tailed *t*-test.

## Data Availability

Not applicable.
